# Spontaneous nosebleeds in the emergency department setting - characteristics, co-morbidities and treatment efficacy

**DOI:** 10.1186/1757-7241-21-S2-A45

**Published:** 2013-09-09

**Authors:** Irfan Rafique, Thomas A Schmidt

**Affiliations:** 1 The Emergency Department, Holbaek University Hospital, Denmark

## Background

Spontaneous nosebleed (SN) is a common cause of visits in Danish emergency departments (ED). The aim of this study was to examine these visits during 1 whole year. We examined the number of visits, characteristics of the patients in terms of sex, age, co-morbidities, and treatment of the SN.

## Methods

Data was extracted from patient records from Holbaek University Hospital. Patients diagnosed with nosebleeds according to the ICD10 classification were sought for in the period 1th October 2011 up to and including 30th September 2012. Nosebleeds due to any kind of trauma were excluded.

## Results

The total number of SNs was 152. Of these 31 were re-visits hence 121 individuals, 54 (45%) female and 67 (55%) male. The age ranged from 2 up to and including 94 years, and age composition was similar in both genders. No patients were found in the age range of 23 up to and including 36. There were 16 (13%) patients below and 105 (87%) above this age range, categorized as younger patients (YP) and older patients (OP), respectively.

YP had no comorbidities and 7 needed conservative treatment using a nasal clamp or putting an ice cube in the mouth.

70 (67%) of the OP had one or more of the following conditions / co-morbidities, using anticoagulant drug treatment (43 patients), hypertension (33 patients), nasal or haematological anomaly (9 patients).

35 (33%) of all OP needed no treatment, 23 (22%) were treated only conservatively and 47 (45%) with RapidRhino®. RapidRhino® was used for 10 (29%) of healthy OP and for 37 (53%) with co-morbidities.

## Conclusion

SN is a condition that frequently appears in EDs. However, no patients were found in the age range 23 up to and including 36, which implies that young adults are not prone to SN. YP needed only conservative treatment, whereas almost half of the OP were treated with a RapidRhino®. If the SN had not ceased spontaneously few OP benefitted from conservative treatment only, especially if they had co-morbidities. This study suggests that conservative treatments in adults are of some benefit, but other means of treatment are mostly necessary.

